# Impact of preoperative docetaxel, cisplatin, and 5-fluorouracil (DCF) therapy on degree of malignant esophageal stenosis

**DOI:** 10.1186/s12876-023-02921-1

**Published:** 2023-08-18

**Authors:** Takahisa Yamaguchi, Koichi Okamoto, Hiroto Saito, Mari Shimada, Toshikatsu Tsuji, Hideki Moriyama, Jun Kinoshita, Keishi Nakamura, Noriyuki Inaki

**Affiliations:** Department of Gastrointestinal Surgery, Kanazawa University, 13-1 Takara-machi, Kanazawa, 920-8641 Japan

**Keywords:** Chemotherapy, Esophageal cancer, Malignant esophageal stenosis, Dysphagia score

## Abstract

**Background:**

Malignant esophageal stenosis is a common and severe complication of advanced esophageal cancer that can be a serious problem in the continuation of chemotherapy and other anticancer treatments. The impact of chemotherapy regimens on the degree of improvement in esophageal stenosis is unknown. In this study, we focused on the impacts of chemotherapy on the direct anticancer effects, and in the improvement of malignant stenosis.

**Methods:**

Patients who underwent radical esophagectomy after chemotherapy, either adjuvant 5-fluorouracil and cisplatin (FP) or docetaxel, cisplatin, and 5-fluorouracil (DCF) regimen, were included. We assessed the length of the cancerous stenosis, the width of the narrowest segment, and the size of the intraluminal area in the stenotic segment by fluoroscopy, and compared the differences before and after chemotherapy. In addition, we evaluated the dysphagia score (Mellow-Pinkas scoring system) as the evaluation of patients’ symptoms. The antitumor effects of chemotherapy were also investigated.

**Results:**

A total of 81 patients were enrolled: 50 were treated with FP, and 31 were treated with DCF. The expansion rate in the length of the narrowest part was significantly increased in the DCF group compared with the FP group. Furthermore, the stenosis index (intraluminal stenotic area/stenotic length) was significantly increased in the DCF group compared with the FP group (112% vs 96%, P = 0.038). Dysphagia score after chemotherapy significantly improved in the DCF group compared to the FP group (P = 0.007). The response rates were 60% in the FP group and 67.7% in the DCF group. Effective histopathological response (improvement to grade 2 or 3) was 24% in the FP group and 38.8% in the DCF group.

**Conclusion:**

DCF therapy is more effective than FP treatment in the improvement of malignant esophageal stenosis.

## Background

Globally, esophageal cancer is one of the most life-threatening diseases and causes approximately 540,000 deaths per year worldwide [1]. Despite recent developments in perioperative management and surgical techniques, esophageal cancer remains a highly lethal malignancy. Surgical treatment is one of the most important therapeutic modalities for esophageal cancer, but it is difficult to improve the outcome of local treatment by surgery alone, and adjuvant chemotherapy is increasingly utilized. The efficacy of neoadjuvant chemotherapy (NAC) for the treatment of esophageal cancer using 5-fluorouracil (5-FU) and cisplatin (FP therapy) has been proven in a phase III Japan Clinical Oncology Group (JCOG) trial (JCOG9907) [2]. Based on this result, preoperative FP therapy has been introduced as the standard treatment for patients with stage II or III esophageal cancer in Japan [3]. The response rate to preoperative FP therapy was only about 38%, however, and the development of a more powerful regimen has been anticipated. In recent years, the results of the JCOG1109 trial confirmed the efficacy of the triple regimen comprising docetaxel, cisplatin, and 5-FU (DCF therapy), and the use of DCF is expected to increase [4].

Malignant esophageal stenosis (MES) is a common and severe complication in patients with locally advanced or unresectable esophageal cancer. MES can cause difficulty in food intake, dysphagia, and aspiration pneumonia, and it can worsen patients’ nutritional status [5–7]. Furthermore, MES affects several aspects of quality of life such as physical experience and the impact on social life [8]. MES can therefore be a serious problem in the maintenance of nutritional status and the continuation of various anticancer treatments. In addition, effective treatment should be selected for patients with reduced quality of life associated with oral intake disorder during chemotherapy.

For these reasons, chemotherapy must be selected with the focus on both the antitumor effect and also the improvement in the stricture and dysphagia. Previously, there were no reports on stenosis improvement in which FP and DCF therapies were compared. In this study, we assessed the relationship between the improvement in MES and chemotherapy regimen in patients who received preoperative FP or DCF therapy for the treatment of esophageal squamous cell carcinoma (ESCC).

## Methods

### Patients

A total of 81 patients who underwent radical esophagectomy after chemotherapy for the treatment of ESCC between January 2008 and April 2015 at Kanazawa university hospital (Kanazawa, Japan) were included. This was a single-center, retrospective cohort study that enrolled patients with resectable cStage II/III ESCC treated with NAC or cStage IVa locally advanced, unresectable ESCC treated with induction chemotherapy. The patients who were received R0 and R1 resection were eligible. All patients had been diagnosed with squamous cell carcinoma (SCC) histologically. Age, sex, histologic type (according to the Lauren classification), TNM stage, and European Cooperative Oncology Group (ECOG) performance status (PS) were evaluated by reviewing medical records. All patients were staged according to the 11th edition of the Japanese Classification of Esophageal Cancer [9]. This study was approved by the Institutional Review Board of Kanazawa University Hospital (study no. 2016 − 289).

### Chemotherapy regimens

From January 2008 to September 2012, all patients were treated with FP as NAC or induction chemotherapy. After September 2012, cStage III and above were treated with DCF. The patients who could not tolerate DCF, elderly patients, and patients with a poor PS were treated with FP by the physician’s choice. The FP regimen included 80 mg/m^2^ of cisplatin on day 1 and 800 mg/m^2^ of 5-FU on days 1–5 for two cycles [2]. The DCF regimen consisted of docetaxel (60–70 mg/m^2^ on day 1), cisplatin (60–70 mg/m^2^, on day 1), and 5-fluorouracil (5-FU; 750–800 mg/m^2^, on days 1–5), for three cycles repeated every 4 weeks [10, 11]. After chemotherapy, all patients were evaluated for resectability and underwent radical thoracoscopic esophagectomy.

### Evaluation of clinical and histopathological response to chemotherapy

The effect of chemotherapy was evaluated according to the Response Evaluation Criteria in Solid Tumors (RECIST), version 1.1 [12]. The clinical response to chemotherapy was classified into one of four categories: complete response (CR), partial response (PR), stable disease (SD), or progressive disease (PD). The histopathological response was categorized into 5 grades according to the evaluation criteria of the Japanese Classification of Esophageal Cancer, 11th edition, [9] as follows: grade 0, no recognizable cytological or histological therapeutic effect; grade 1a, viable cancer cells accounting for two-thirds or more of the tumor tissue; grade 1b, viable cancer cells accounting for one-third or more but less than two-thirds of the tumor tissue; grade 2, viable cancer cells accounting for less than one-third of the tumor tissue; and grade 3, no viable cancer cells.

### Assessment of stenosis before and after chemotherapy

**Fig. 1:**
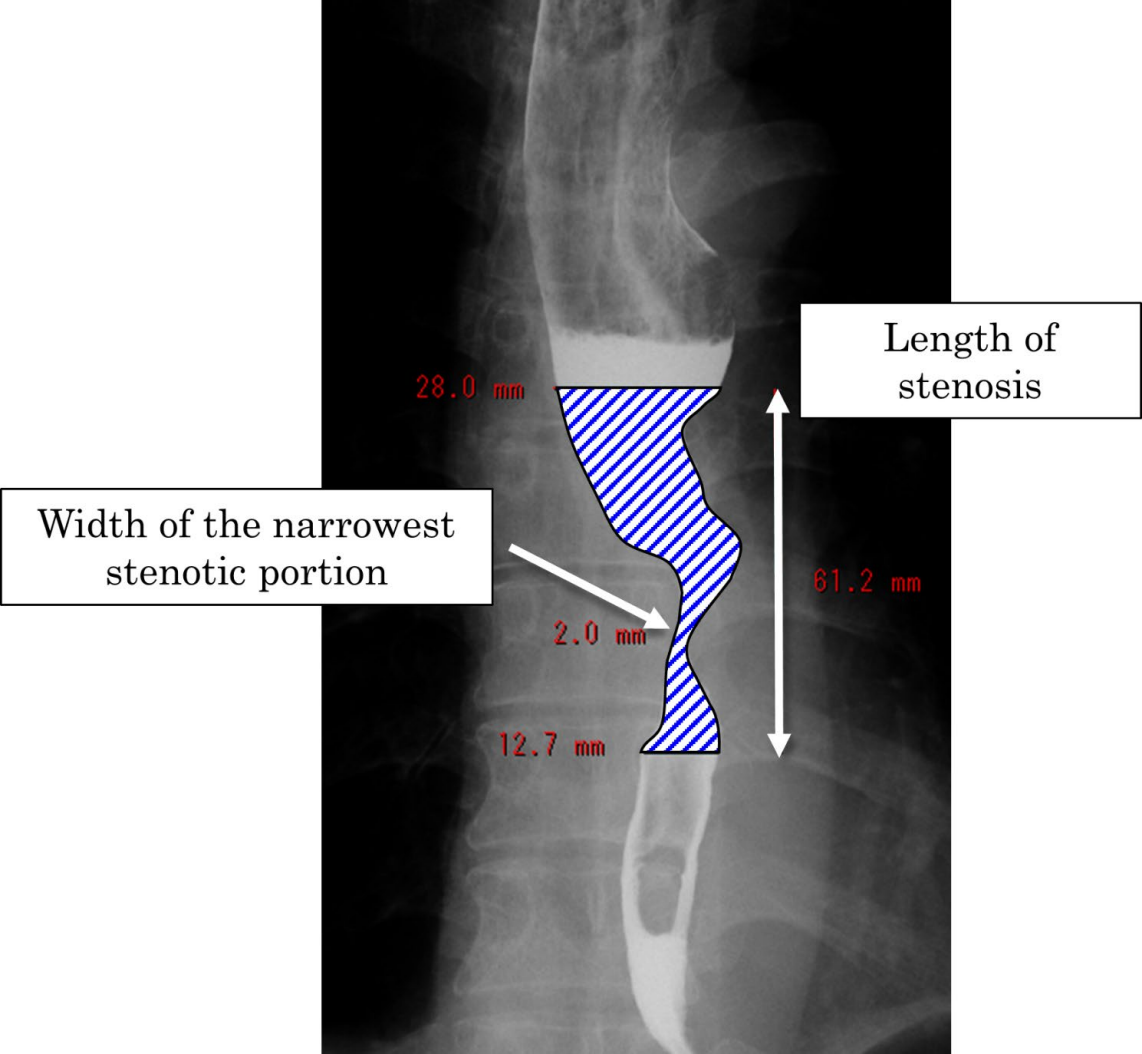
Representative schema of the esophageal stenosis measurement using esophageal fluoroscopy. The shaded area was defined as the intraluminal stenotic area

We assessed the stenotic portion, the width of the narrowest stenotic portion, and the intraluminal area of the cancerous stenosis before and after chemotherapy by manual trace measuring of a digital image from esophageal fluoroscopy. The beginning and the end of the stenosis were defined as the area where the wall irregularities or changes were observed by fluoroscopy. A representative schema is shown in Fig. 1. In addition, we used the stenosis index (SI) as an indicator of the degree of stenosis. The SI was calculated by dividing the intraluminal stenotic area by the length of stenosis (intraluminal stenotic area/length of stenosis).

### Dysphagia score before and after chemotherapy

The ability to swallow was assessed as a dysphagia score based on symptoms tumoral stricture. Dysphagia scores were retrieved from patients’ medical records before and after chemotherapy according to Mellow and Pinkas’ scoring system [13], as follows: 0, able to eat a normal diet; 1, able to eat some solid food; 2, able to eat semi-solid food only; 3, able to swallow liquids only; and 4, complete dysphagia. A response was defined as an improvement of dysphagia score from the baseline with at least − 1 level.

### Data analysis

The χ^2^, Fisher’s exact, Mann–Whitney *U*, and Student’s *t* tests were used to compare the categorical variables. A P-value < 0.05 was considered a statistically significant difference. All statistical analyses were performed using IBM SPSS Statistics for Windows, version 23.0 (IBM Corp., Armonk, NY, USA).

## Results

### Relationship between chemotherapy regimen and clinicopathological characteristics

The clinicopathological characteristics of the patients who received FP or DCF therapy are shown in Table 1. Fifty patients (61.7%) received the FP regimen, and 31 patients (38.3%) received the DCF regimen as NAC or induction chemotherapy. In univariate analysis of clinical variables, age, sex, tumor location, pathology, PS, pT, pN, and pStage were not significantly associated with the chemotherapeutic regimen (Table 1). The length of the long axis of the primary tumor was significantly longer in the DCF group than in the FP group (P = 0.005). Clinical stage was more advanced in the DCF group than in the FP group (P = 0.048, respectively).

**Table 1:** Characteristics of patients treated with FP or DCF therapy

		FP group (n = 50)	DCF group (n = 31)	*P* value
Age	Median (range)	64 (48–76)	63 (47–75)	0.137 ^a^
Sex	male / female	41 / 9	25 / 6	0.879 ^b^
Performance status	0 / 1	47 / 3	29 / 2	0.653 ^c^
Tumor location	Ce / Ut / Mt / Lt / Ae	2 / 4 / 29 / 14 / 1	1 / 5 / 11 / 13 / 1	0.359 ^b^
The degree of differentiation	well / moderate / poorly / unknown	7 / 20 / 18 / 5	4 / 14 / 8 / 5	0.720 ^b^
Tumor length in major axis (cm)	Mean ± SD	5.3 ± 2.5	7.3 ± 3.7	*0.005 ^a^
cStage (11th JES)	II / III / IVa	17 / 20 / 13	3 / 17 / 11	*0.048 ^b^
pT (11th JES)	0-1a / 1b / 2 / 3 / 4	4 / 11 / 5 / 26 / 4	6 / 2 / 5 / 14 / 4	0.199 ^b^
pN (11th JES)	0 / 1 / 2 / 3 / 4	17 / 4 / 13 / 6 / 10	7 / 2 / 12 / 3 / 7	0.786 ^b^
pStage (11th JES)	I / II / III / IVa	4 / 14 / 19 / 13	2 / 5 / 12 / 12	0.529 ^b^

FP, 5-fluorouracil and cisplatin; DCF, docetaxel, cisplatin, and 5-fluorouracil; 11th JES, 11th edition of the Japanese Classification of Esophageal Cancer; Ce, cervical esophagus; Ut, upper thoracic esophagus; Mt, middle thoracic esophagus; Lt, lower thoracic esophagus; Ae, abdominal esophagus; SD, standard deviation;

*p < 0.05

^a^ Student’s *t*-test; ^b^ Fisher’s exact test; ^c^ χ^2^ test

### Clinical and histopathological response to preoperative chemotherapy

The clinical response and histopathological therapeutic effects after NAC are shown in Table 2. The response rates (CR + PR) using RECIST was 60% in the FP group and 67.7% in the DCF group, with no significant difference between the two groups. On the other hand, the endoscopic response rate in DCF group was significantly more effective than that in FP group (P = 0.040). The effective histopathological response, defined as grade 2 or 3, was 24% in the FP group and 38.8% in the DCF group. Furthermore, the patients in the DCF group tended to have a more favorable response (P = 0.214), though no statistically significant difference was found.

**Table 2:** Clinical and histopathological response to preoperative FP or DCF therapy

	FP group (n = 50)	DCF group (n = 31)	*P* value
Overall response (RECIST)			
CR	4 (8.0)	3 (9.7)	
PR	26 (52.0)	18 (58.1)	
SD	13 (26.0)	7 (22.6)	
PD	7 (14.0)	3 (9.7)	
Response rate (CR + PR)	30 (60.0)	21 (67.7)	0.483 ^a^
Endoscopic response rate (CR + PR)	28 (54.9)	24 (77.4)	*0.0401^a^
Histopathological response in surgically resected cases			
Grade 0	6 (12.0)	7 (22.6)	
Grade 1a	30 (60.0)	12 (38.7)	
Grade 1b	4 (8.0)	2 (6.5)	
Grade 2	8 (16.0)	8 (25.8)	
Grade 3	2 (4.0)	2 (6.5)	
Grade ≥ 2	10 (20.0)	10 (32.3)	0.214 ^a^

FP, 5-fluorouracil and cisplatin; DCF, docetaxel, cisplatin, and 5-fluorouracil; RECIST, Response Evaluation Criteria in Solid Tumors; CR, complete response; PR, partial response; SD, stable disease; PD, progressive disease;

*p < 0.05

^a^ χ^2^ test

### Assessment of MES before and after chemotherapy on esophageal fluoroscopy

We assessed the degree of MES before and after chemotherapy on esophageal fluoroscopy, and the results are shown in Table 3. The average length of the cancerous stenosis was significantly longer in the DCF group than in the FP group before NAC. Furthermore, the average width of the narrowest portion of the cancerous stenosis was significantly shorter in the DCF group before chemotherapy. These results indicate that the primary tumors in the DCF group were larger and narrower than in the FP group. The reduction rate in the stenotic length did not significantly differ between the two groups, but the expansion rate in the width of the cancerous stenosis was significantly increased in the DCF group (DCF 127% vs. FP 102%, P = 0.031). Even if the intraluminal area of the stenosis decreases, the length of the stenosis may also decrease at the same time. We thought that the SI (intraluminal stenotic area/length of stenosis) is important as an indicator of the essential severity of MES. The SI before and after chemotherapy was not different in either group. However, the rate of SI change was significantly increased in the DCF group compared with the FP group (DCF 112% vs. FP 96%, P = 0.038), indicating improvement in the severity of MES.

**Table 3:** Assessment of malignant esophageal stenosis before and after chemotherapy

			FP group (n = 50)	DCF group (n = 31)	*P* value
Length of stenosis (cm, mean ± SD)		Before	4.6 ± 1.74	6.0 ± 2.06	*0.003 ^a^
		After	4.3 ± 1.55	5.3 ± 1.94	*0.012 ^a^
Reduction rate in the stenotic length (%, mean)			6.6	10.8	0.298 ^a^
Width of the narrowest portion (cm, mean ± SD)		Before	1.1 ± 0.59	0.8 ± 0.44	*0.030 ^a^
		After	1.0 ± 0.55	1.0 ± 0.45	0.862 ^a^
Expansion rate in the width of narrowest portion (%, mean)			102	127	*0.031 ^a^
Intraluminal stenotic area (cm2, mean ± SD)		Before	6.5 ± 2.73	8.3 ± 3.90	*0.034 ^a^
		After	5.6 ± 2.40	7.8 ± 4.07	*0.009 ^a^
Stenosis lumen area reduction rate (%, mean)			11.1	2.6	0.176 ^a^
SI (mean)		Before	1.47 ± 0.48	1.38 ± 0.39	0.392 ^a^
		After	1.37 ± 0.47	1.47 ± 0.42	0.353 ^a^
The rate of SI change (%, mean)			96	112	*0.038 ^a^

Numbers given as n (%)

SD, standard deviation; SI, stenosis index (intraluminal stenotic area/length of stenosis); FP, 5-fluorouracil and cisplatin; DCF, docetaxel, cisplatin, and 5-fluorouracil;

*P < 0.05

^a^ Student’s *t*-test

### Improvement of dysphagia score in FP and DCF group

We assessed the correlations between the improvement of dysphagia score and chemotherapy regimen (Table 4). Dysphagia score before chemotherapy tended to be worse in the DCF group, though no statistically significant difference was found. On the other hand, the dysphagia score of the patients in the DCF group significantly frequently improved than that of the FP group after chemotherapy (P = 0.007).

**Table 4:** The dysphagia score in the FP and the DCF group

		FP group (n = 50)	DCF group (n = 31)	*P* value
The dysphagia score before chemotherapy	0–2 / 3–4	41 / 9	21/10	0.141 ^a^
The improvement of the dysphagia score	Yes / No	14 / 36	18 / 13	*0.007^a^

FP, 5-fluorouracil and cisplatin; DCF, docetaxel, cisplatin, and 5-fluorouracil;

*p < 0.05

^a^ χ^2^ test

### Factors predicting the rate of SI change

We assessed the correlation between the rate of SI change and clinicopathological features. Patients in the median proportion were divided into two groups (SI low or SI high), according to the value of the SI. The median proportion of the rate of SI change rate was 99%, and we used this value as the cut-off. The relationship between the rate of SI change and clinicopathological valuables is shown in Table 5. There was no significant relationship between the rate of SI change and age, sex, tumor location, tumor size, stage, or RECIST score. The DCF group was significantly related to a high rate of SI change (P = 0.024). In addition, histopathological response of grade 2 or 3 tended to have high rate of SI change (P = 0.061).

**Table 5:** The predicting factors of the rate of SI change

		SI low group (n = 39)	SI high group (n = 42)	*P* value
Age	≥ 70 yrs. / 70 yrs.>	9 / 30	11 / 31	0.745 ^a^
Sex	male / female	30 / 9	36 / 6	0.309 ^a^
Tumor location	Ce / Ut / Mt / Lt / Ae	1 / 2 / 20 / 14 / 2	2 / 7 / 20 / 13 / 0	0.283 ^a^
cStage (11th JES)	II / III / IVa	9 / 20 / 10	11 / 17 / 14	0.606 ^a^
pStage (11th JES)	I / II / III / IVa	3 / 9 / 15 / 12	3 / 10 / 16 / 13	> 0.999 ^a^
Overall response	SD + PD / PR + CR	29 / 10	27 / 15	0.327 ^a^
Histopathological response	0 and 1 / 2 and 3	33 / 6	28 / 14	0.061 ^a^
Regimen	FP / DCF	29 / 10	21 / 21	*0.024 ^a^

11th JES, 11th edition of the Japanese Classification of Esophageal Cancer; Ce, cervical esophagus; Ut, upper thoracic esophagus; Mt, middle thoracic esophagus; Lt, lower thoracic esophagus; Ae, abdominal esophagus; CR, complete response; PR, partial response; SD, stable disease; PD, progressive disease; FP, 5-fluorouracil and cisplatin; DCF, docetaxel, cisplatin, and 5-fluorouracil; SI, stenosis index (intraluminal stenotic area/length of stenosis);

*P < 0.05

^a^ χ^2^ test

## Discussion

In this study, we investigated the relationship between the chemotherapy regimen and its impact on MES. Our data showed that DCF therapy is effective in the improvement of MES. Recently, the efficacy of DCF therapy for esophageal cancer has been described by Watanabe et al., who reported that the clinical response rate to DCF therapy was 53%, and pathological response rate was 36% for preoperative chemotherapy as NAC [14]. Other reports showed that the OS of ESCC was significantly longer for DCF therapy than for FP therapy, as well as RFS [4, 15]. In addition, DCF therapy as induction chemotherapy for initially unresectable, locally advanced esophageal cancer elicits a good response and improves the prognosis. The DCF regimen was superior to the FP regimen with regard to OS, R0 resection rate, and histopathological response rate [7, 16]. However, the JCOG1109 trial was adapted for patients up to 75 years old, and its high frequency of adverse events raises the question of whether it should be given as NAC to all operable patients with advanced esophageal cancer [4]. Therefore, it is necessary to consider the benefits and potential adverse effects of DCF therapy in addition to the improvement in prognosis.

The expansion rate in the narrowest width after chemotherapy was significantly increased in the DCF group compared with the FP group. In addition, the SI index, which is thought to represent the severity of MES, was significantly improved in the DCF group compared with the FP group. These results indicate that the DCF regimen is effective not only for its reductive anticancer effect, but also for the improvement of MES. To our knowledge, this is the first study to report the improvement of MES and the type of chemotherapy.

Furthermore, we assessed the dysphagia score before and after chemotherapy as the evaluation of patients’ symptoms. DCF regimen was also found to be effective in the improving of patients’ dysphagia scores. This indicates that DCF therapy improves patients’ intake disorder, and is clinically useful for patients with MES.

Dysphagia resulting from MES leads to malnutrition and difficulty continuing chemotherapy [17–19], so it is important to reduce the dysphagia and improve the nutrition status. Esophageal stent implantation was useful and immediate for the palliative treatment for MES [20–24]. However, esophageal stents can have complications, such as bleeding, migration into the stomach or small bowel, aspiration pneumonia, and fistula formation [25–27]. In addition, the oncologic safety and the effectiveness of the esophageal stent in patients with non-palliative esophageal cancer are not well known. The European Society of Gastrointestinal Endoscopy has not recommended the temporary placement of a self-expandable metallic stent (SEMS) for MES as a bridge to surgery or before preoperative chemoradiotherapy [28]. The choice of chemotherapy is important because SEMS should not be used for patients with ESCC before surgery.

In the analysis of the two groups with respect to the rate of SI change, a high level of SI change was identified; that is, the improvement of MES, is significantly correlated with patients who only received DCF therapy, though there were no significant changes in other clinicopathological features including tumor stages. There is a possibility that the hardness and degree of stenosis of the primary tumor may be related to the amount of stromal fibrosis in the tumor tissue. DCF therapy may suppress the stromal fibrosis and improve the solidity and degree of stenosis of the primary tumor.

Taxane-based chemotherapy has been commonly used for the treatment of several cancers such as those of the stomach, esophagus, breast, and ovary. Paclitaxel (PTX), one of the major taxane drugs, improves intestinal stenosis related to the cancer-associated fibrosis in patients with gastric cancer and peritoneal metastasis [29]. In this study, the DCF group using Docetaxel showed a significant improvement in the width of the narrowest portion and SI, suggesting that the tissue softened and expanded better on examination by esophageal fluoroscopy. These results indicate that taxane-containing chemotherapy may be suppressing not only the cancer progression but also the tumor fibrosis and MES. However, there have been a few reports on the inhibition of fibrosis by docetaxel, another taxane anticancer drug.

Docetaxel is a microtubule-stabilizing taxane, and it has increased affinity for tubulin [30]. This drug suppresses the expression of TGF-β, as does paclitaxel [31]. Therefore, it is thought that docetaxel may inhibit the function of fibroblasts and fibrocytes that are associated with stromal fibrosis in cancer tissues. The details, however, are not yet known. Further in vitro and in vivo studies on docetaxel are needed.

Though DCF therapy is effective in the improvement of MES, there was no significant change in the prognosis between the FP and DCF groups in our study (data not shown). Patients with more advanced diseases were included in the DCF group, which may have affected the treatment outcomes and prognosis. The results of JCOG 1109 showed a significant survival benefit in the DCF group, making it a highly effective treatment as NAC for the patients with resectable esophageal cancer (median survival time: FP 5.6 years, DCF 6.7 years, *p* = 0.006) [4]. However, adverse events such as neutropenia and anorexia have occurred more frequently in DCF therapy. Therefore, we have to use this regimen in consideration of the patient comorbidities, age, condition, and degree of esophageal cancer. The results of our study suggest that DCF therapy should be recommended for the patients with ESCC and MES.

This study has some limitations. First, this investigation was retrospective in nature and was conducted at a single institution. The number of cases is small and there are differences in patient backgrounds between the FP and the DCF groups. Second, we did not evaluate the degree of fibrosis at the tumor site in either group. The assessment of fibrosis using Azan staining and alpha-smooth muscle actin immunohistochemical staining may provide valuable data, and further the understanding of the mechanism of stenosis improvement in ESCC. These factors should be considered, and further prospective, multicenter studies are needed to confirm our results.

## Conclusions

Our study showed that the DCF regimen was more effective for improving MES than the FP regimen. We suggest that DCF therapy should be selected more frequently for resectable ESCC with MES to improve patients’ intake disorder and quality of life.

## Data Availability

The datasets used during the current study available from the corresponding author on reasonable request.

## Abbreviations

DCF: Docetaxel, cisplatin, and 5-fluorouracil
FP: 5-fluorouracil and cisplatin
NAC: Nneoadjuvant chemotherapy
MES: Malignant esophageal stenosis
ESCC: Esophageal squamous cell carcinoma
SCC: Squamous cell carcinoma
RECIST: Response Evaluation Criteria in Solid Tumors
CR: Complete response
PR: Partial response
SD: Stable disease
PD: Progressive disease
SI: Stenosis index
